# SARS-CoV-2: One Year in the Pandemic. What Have We Learned, the New Vaccine Era and the Threat of SARS-CoV-2 Variants

**DOI:** 10.3390/biomedicines9060611

**Published:** 2021-05-27

**Authors:** Filippo Scialo, Maria Vitale, Aurora Daniele, Ersilia Nigro, Fabio Perrotta, Monica Gelzo, Carlo Iadevaia, Francesco Saverio Cerqua, Adriano Costigliola, Valentino Allocca, Felice Amato, Lucio Pastore, Giuseppe Castaldo, Andrea Bianco

**Affiliations:** 1 Dipartimento di Scienze Mediche Traslazionali, University of Campania “L. Vanvitelli”, 80131 Naples, Italy; andrea.bianco@unicampania.it; 2 CEINGE, Biotecnologie Avanzate, 80131 Naples, Italy; vitalema@ceinge.unina.it (M.V.); aurora.daniele@unicampania.it (A.D.); nigro@ceinge.unina.it (E.N.); monica.gelzo@unina.it (M.G.); felice.amato@unina.it (F.A.); giuseppe.castaldo@unina.it (G.C.); 3 Dipartimento di Medicina Molecolare e Biotecnologie Mediche, Università di Napoli Federico II, 80131 Naples, Italy; 4 Dipartimento di Scienze e Tecnologie Ambientali Biologiche Farmaceutiche, University of Campania “L. Vanvitelli”, 80131 Naples, Italy; 5 U.O.C Pneumologia Azienda Ospedaliera Sant’Anna e San Sebastiano, 81100 Caserta, Italy; dott.fabioperrotta@gmail.com; 6 Pneumology Vanvitelly-COVID Unit A.O. dei Colli Hospital Monaldi, 80131 Naples, Italy; dott.carlo.iadevaia@gmail.com (C.I.); fscerqua@gmail.com (F.S.C.); neon17ster@gmail.com (A.C.); valentino.allocca@unicampania.it (V.A.)

**Keywords:** COVID19, SARS-CoV-2, coronavirus, ACE2, renin angiotensin aldosterone system, kinin-kallikrein system

## Abstract

Since the beginning of 2020, the new pandemic caused by SARS-CoV-2 and named coronavirus disease 19 (COVID 19) has changed our socio-economic life. In just a few months, SARS-CoV-2 was able to spread worldwide at an unprecedented speed, causing hundreds of thousands of deaths, especially among the weakest part of the population. Indeed, especially at the beginning of this pandemic, many reports highlighted how people, suffering from other pathologies, such as hypertension, cardiovascular diseases, and diabetes, are more at risk of severe outcomes if infected. Although this pandemic has put the entire academic world to the test, it has also been a year of intense research and many important contributions have advanced our understanding of SARS-CoV-2 origin, its molecular structure and its mechanism of infection. Unfortunately, despite this great effort, we are still a long way from fully understanding how SARS-CoV-2 dysregulates organismal physiology and whether the current vaccines will be able to protect us from possible future pandemics. Here, we discuss the knowledge we have gained during this year and which questions future research should address.

## 1. Introduction

The last two decades have been punctuated by the sudden appearance of viruses able to quickly spread among continents and cause large-scale pandemics, such as SARS-CoV in 2003, MERS-COV in 2012, and SARS-CoV-2 since the beginning of 2020 [1]. At present, one year from the start of the COVID-19 pandemic, the efforts of the academic world and pharmaceutical industry have resulted in the development of better therapies and the production of multiple vaccines that hold promise for a better future. Unfortunately, despite this great effort, we are still far from fully understanding the mechanisms that SARS-CoV-2 uses to dysregulate many physiological pathways causing hyperactivation of the immune response and multiorgan dysfunction [2]. In fact, COVID-19 is not only a lung disease, and unravelling the molecular pathways dysregulated by SARS-CoV-2 in different organs will help to develop specific therapies [3]. Moreover, the appearance of new SARS-CoV-2 variants is constantly raising the question of whether the currently available vaccines have the potential to prevent possible future pandemics. Here, we first discuss what we have learned about SARS-CoV-2’s origin, mechanism of infection, and how its sequence has changed, collecting the available information about the impact of SARS-CoV-2 new variants. We will then focus on the main role that the dysregulation of its receptor ACE2 plays in determining disease progression, especially in the organs that seem to be more affected. We will discuss how the dysregulation of the Renin–angiotensin aldosterone system (RAAS) and Kinin–Kallikrein system (KKS) [4] can, in part, explain the overproduction of proinflammatory cytokines and the coagulopathy seen in severe cases of COVID-19. Furthermore, we will describe the available therapies offered at present, and how the advancement in our understanding of SARS-CoV-2 infection is leading to the development of new treatments. Finally, we will discuss how the current vaccination campaign taking place worldwide is affecting the rate of infection and death, especially among the weakest part of the population.

## 2. SARS-CoV-2 Origin and Molecular Structure

**Figure 1:**
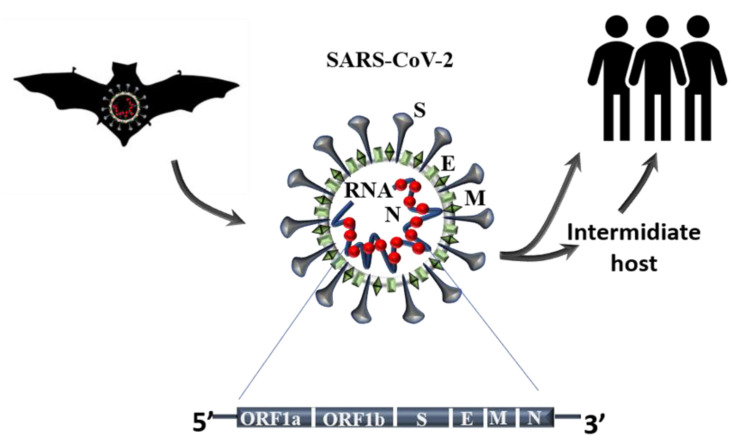
SARS-CoV-2 origin and molecular structure. The natural reservoir of the new betacoronavirus Sars-CoV-2 has been demonstrated to be bats and thought to spread to humans through an intermediate host. The viral RNA is associated with the N proteins that are involved in the key process of infection such as transcription, replication, and packaging. The lipid membrane that protects the viral RNA contains structural proteins such as membrane (M) and envelope proteins (E). The spike glycoprotein (S), through its receptor-binding domain, is responsible for the recognition of the host cell receptor. The picture shows a simplification of the viral genome.

Coronaviruses (CoV) are enveloped positive-stranded RNA viruses belonging to the Coronaviridae family. Four members of this family—CoV-NL63, -229E, -OC43, and -HKU1—have already been identified in humans and known to cause endemic mild respiratory tract infection, although fatal outcomes have been reported, particularly in immunocompromised patients [5]. In the last twenty years, three newly identified members of this family have been responsible for causing epidemic severe acute respiratory syndrome (SARS), from which they have been named SARS-CoV, middle east respiratory syndrome (MERS), and SARS-CoV-2 [6,7], the latter of which is responsible for the ongoing outbreak. Several studies have demonstrated that the natural reservoir host for these viruses is bats [8]; usually, infection of an intermediate species occurs prior to transmission to humans (Figure 1).

To date, all evidence suggests that SARS-CoV-2 is the result of an infection from an intermediate host and not a result of laboratory manipulation [8]. In fact, since the first outbreak in 2002, a considerable number of SARS-like viruses have been identified in bats and shown to have the capacity to infect humans [7], supporting the hypothesis that potential future outbreaks are possible. The diversity in the coronavirus family is generally believed to be due to the lack of proofreading activity of the RNA-dependent RNA polymerase (RdRp) which is necessary for viral RNA replication. Recent data have instead shown that RdRp of both SARS-CoV and SARS-CoV-2 has proofreading activities and a decrease in the replication fidelity is probably due to mutations in a specific exon [9]. Indeed, although SARS-CoV-2 is closely related to SARS-CoV, sharing 96% of its identity (Table 1), genomic analysis has demonstrated that mutations in the spike glycoprotein of SARS-CoV-2 increased the affinity for its receptor ACE2 [8].

## 3. The Effect of Sars-Cov-2 New Variants

Structurally, the spike glycoprotein is formed by two subunits, named S1 and S2. The S1 subunit is responsible for receptor binding and can be further divided into N terminal-subunit (NTD) and the receptor-binding domain (RBD). The S2 subunit is necessary for the membrane fusion between the virus envelop and the late endosome membrane. Moreover, the genomic structure of the viral RNA, 2.9kb in length [10], contains genes necessary for its replication, such as the RdRp and helicase (HEL), genes encoding structural proteins such as membrane (M), nucleocapsid (N) and envelope protein (E) and, as we discussed above, the spike glycoprotein (S), responsible for host recognition [8].

**Figure 2:**
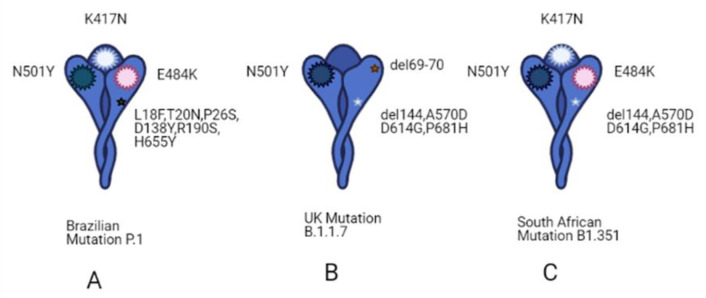
Schematic representation of spike variants. (**A**) Brazilian mutation called P.1, that shares three mutations in the RBD domain of spike protein with South African variants (N501Y, E484K and K417T); P.1 has 17 amino acid changes, nine of which are in its spike protein (L18F, T20N, P26S, D138Y, R190S, H655Y). (**B**) English mutation called B1.1.7 has a mutation (N501Y) in the RBD of the spike protein like P.1 and B1.351 variants. Additionally, amino acid deletions were found within the N-terminal domain (NTD) of spike protein, important for efficient entry into host cells. (**C**) South African mutation called B1. 351, shows a mutation in spike protein (N501Y, E484K and K417T) and several changes in NTD spike domain (A570D, D614G, P681H), including amino acid deletion (del144). [14] Created with BioRender.com.

In the months of 2020, more attention was focused on the identification of new SARS-CoV-2 variants, some of which have caused an unexpected increase in COVID-19 cases. For instance, the variant 501Y.V1 (B.1.1.7), known also as the English variant, has been associated with an increased infectivity and high transmission through populations, possibly due to an enhanced affinity for its receptor ACE2. At the same time, in South Africa, another variant called 501Y.V2 (B1.351) is spreading widely through the population [11,12,13]. These two variants share a mutation in the RBD of the spike protein, with the South African variant having two additional mutations, E484K and K417N, that allow the immune escape of SARS-CoV-2 and, in particular, to the neutralizing antibodies [12,13]. Another variant identified in Brazil (P.1 (501Y.V3)), shows three mutations in the RBD, shared with south African variants, but the infectivity rate of this variant is still under investigation (Figure 2). At this point, the most relevant issue is whether the available COVID-19 vaccines will be able to protect the population from these and new variants that will be identified. It is worth remembering that the three main vaccines, Pfizer BioNTech, Moderna, and Oxford AstraZeneca, which have already been administered to millions of people, target the spike protein. Some tests are underway to evaluate their effectiveness against these variants, even if the spike protein is a large protein and a lot of mutations would be needed to completely escape the immune system [11].

## 4. SARS-CoV-2 Mechanism of Infection and Immune Response Activation

### 4.1. Mechanism of Infection

**Figure 3:**
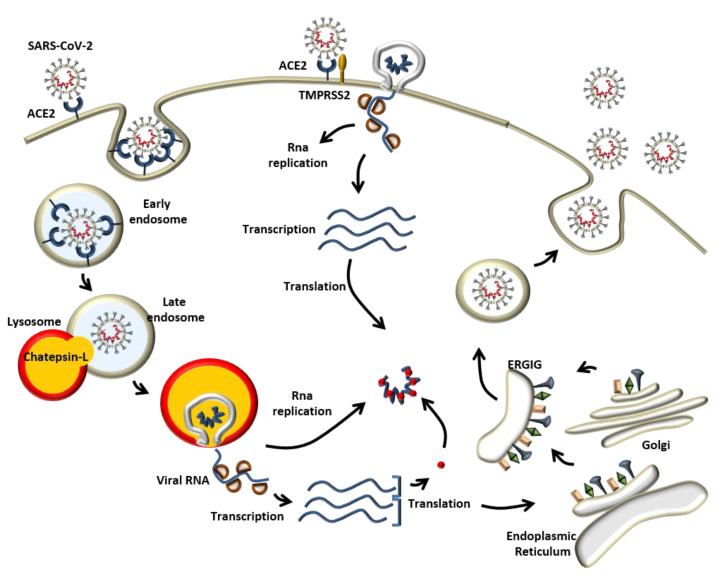
Mechanism of infection. SARS-CoV-2 recognizes the host cell by binding with the Angiotensin- converting enzyme 2 (ACE2) via Spike glycoprotein S1 unit. The priming of the Spike glycoprotein can be mediated by the TMPRSS2 protease that allows virus/membrane fusion guided by the S2 unit. Alternatively, the virus can enter the cell by using the endocytic pathway where Chatepsin-L cleaves the S protein allowing also in this case the priming of the late endosome membrane with the S2 unit. The viral RNA will undergo transcription and replication. The new viral particle will be built in the ER-Golgi intermediate compartment (ERGIC) and released by exocytosis.

Experimental evidence has demonstrated that ACE2 is used by SARS-CoV-2 as the main entry point into the cells [1,14,15]. As described above, the S1 unit of SARS-CoV-2 S protein is responsible for binding with the host cell receptor ACE2. Here, the S protein can be cleaved by the protease TMPRSS2 [16] between S1/S2 units, allowing for direct fusion with the cellular membrane mediated by the S2 unit (Figure 3). Alternatively, after binding with ACE2, SARS-CoV-2 can take advantage of the endocytic pathway to enter the cells (Figure 3). Apilimod and YM201636, two potent inhibitors of phosphatidylinositol-3,5-bisphosphate (PI (3,5) P2), which controls endosome dynamics, have been shown to greatly reduce SARS-CoV-2 entry. Upon fusion with the lysosome, cathepsin-L will drive the proteolytic cleavage of the Spike glycoprotein activating the S2 subunit and the consequent membrane fusion to release the viral RNA into the cytoplasm [17]. Here, the viral RNA will be translated to form RdRp and Hel, important for its transcription and replication. Structural proteins will be transcribed, translated in the endoplasmic reticulum, and transported to the Golgi. The new viral RNA with the structural proteins will be used to build a new virus in the ER-Golgi intermediate compartment (ERGIC), which is released by exocytosis and ready to infect other cells (Figure 3). Although ACE2 has been demonstrated to be the major receptor for SARS-CoV-2, the intense research into its mechanism of infection coupled with the screening of compounds to reduce its entry into the cells, has led to the identification of other receptors that could possibly play a role in the severity of the disease and explain how this virus is able to target multiple organs. For instance, Meplazumab, an anti-CD147 antibody, has been demonstrated to inhibit SARS-CoV-2 infection in Vero cells [18]. CD147 receptor belongs to the immunoglobulin superfamily and, in contrast to ACE2, is mainly expressed in the brain [19], which could, in part, explain the neurological problems in COVID-19 patients. Neuropilin 1 (NRP1) has also been shown to bind the SARS-CoV-2 Spike protein [20] and, since it is abundantly expressed in the epithelial cells of the olfactory bulb, this could also explain the high rate of infectivity of this virus.

### 4.2. Immune Response Activation

**Figure 4:**
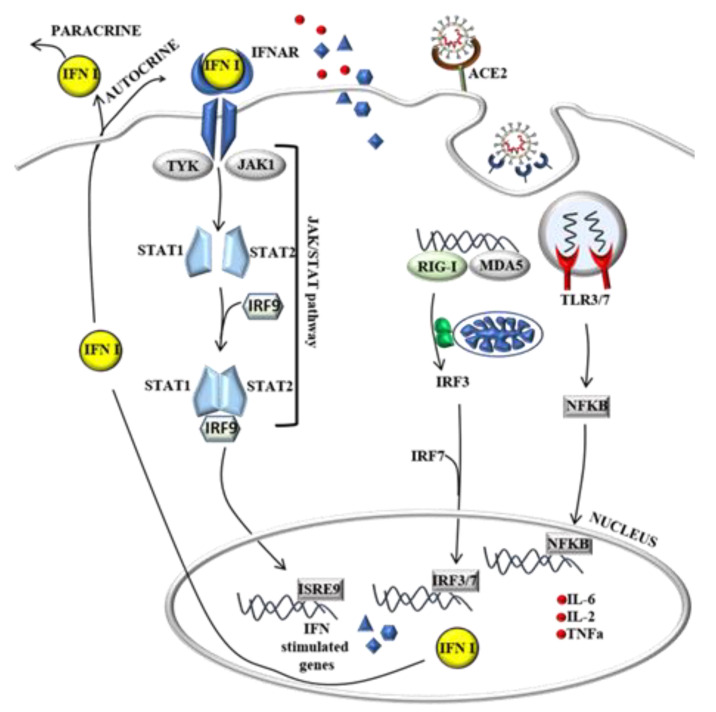
Immune response activation due to SARS-CoV-2 infection. The presence of virus particles in the cell, such as the viral RNA, is recognized by the Toll-Like and RIG-I/MAD5 pathway and will initiate signaling cascades resulting in the translocation of NF-kb and IRF3/7 into the nucleus and the transcription of pro-inflammatory cytokines that are responsible for recruiting immune cells to the site of infection (left).

Laboratory abnormalities of most affected COVID-19 patients include neutrophilia, lymphopenia, increased C- reactive protein, and pro-inflammatory cytokines such as interleukin 6 (IL-6), IL-2, IL-7 and tumor necrosis factor-alpha (TNFα) among others [21]. The activation of the immune response starts with the recognition of virus components generally called pathogen-associated molecular patterns (PAMPs), such as single-strand RNA (ssRNA) or double-strand RNA (dsRNA), formed as an intermediate during RNA replication. ssRNA and dsRNA can be identified by the cellular pattern recognition receptors (PRRs) such as the Toll-Like (TLR) and RIG-I/MDA5 pathway, respectively (Figure 4). The latest scientific findings have demonstrated that MDA5 is the major sensor involved in the recognition of SARS-CoV-2. The infection of different model systems such as lung epithelial cells CALU-3, intestinal cells CACO-2 and nasal epithelial-derived cells has been shown to induce a strong expression of MDA5 upon SARS-CoV-2 infection, together with other viral RNA sensors such as LGP2 and NOD1 [22,23,24]. The activation of the endosomal TLR3/7 and RIG-I/MDA5 pathway will then initiate a signalling cascade that results in the translocation of the NF-kb and Interferone Regulatory 3/7 transcription factor (IRF3/7) into the nucleus and the transcription of pro-inflammatory cytokines and chemokines such as TNFα, IL-6 and Interferon 1 (IFN1). IFN1 is then transported out of the cells to bind the IFNAR receptor and guide the transcription of more IFN1 and IFN-stimulated genes that, in combination with the cytokine gradient, are necessary to recruit neutrophils, macrophages, and other immune cells to the site of infection (Figure 4). One of the problems is that SARS-CoV-2 might have the ability to inhibit IFN1 production [25]. In fact, in the same model system described previously, it has also been shown that SARS-CoV-2 infection induced a delayed interferon response compared with other viruses [22,23]. Two interesting works have demonstrated that SARS-CoV-2 can operate this inhibition through different molecular mechanisms. One is represented by the papain-like protease activity of the gene NSP3 that has been demonstrated to inhibit the ISGylation of MDA5, which is important for its activation, delaying, in this way, the Interferon Stimulated Gene (ISG) response [26]. The second mechanism relies on the ability to inhibit the IRF3/7 nuclear translocation operated by the SARS-CoV-2 NSP12 gene that encodes the RdRp [27].

## 5. Dysregulation of RAAS and KKS as a Probable Cause of the Immune Response Overactivation

**Figure 5:**
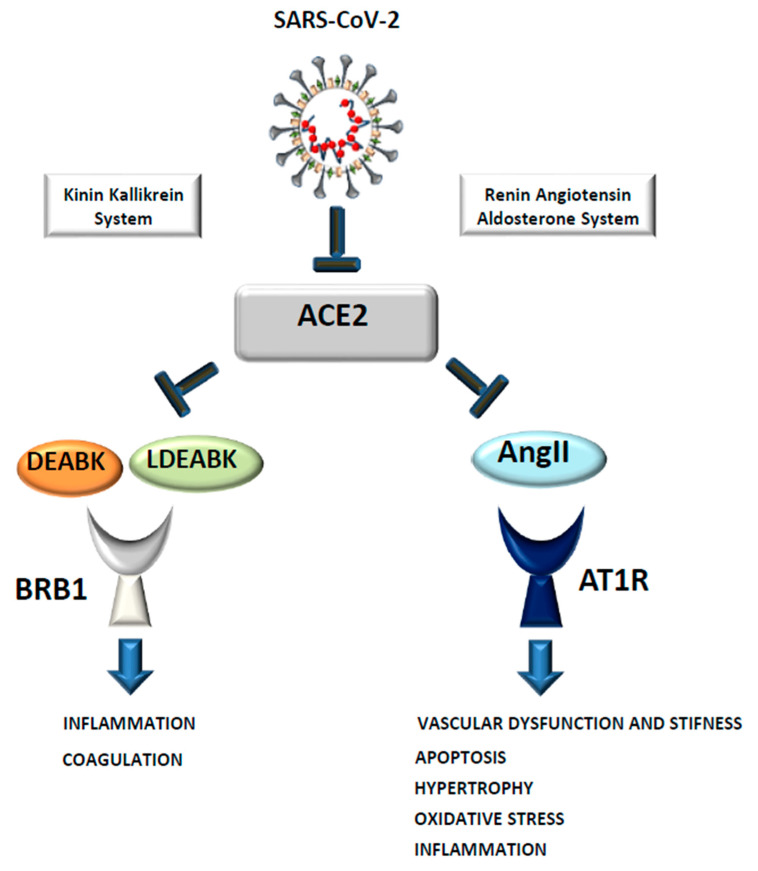
SARS-CoV-2 dependent ACE2 internalization as a possible cause of the cytokine storm. The binding of SARS-CoV-2 to ACE2 can cause its internalization and a decreased level on the plasma membrane. This leads to an increase in AngII and DEABK/LDEABK causing vasoconstriction, apoptosis, oxidative stress, and an overproduction of proinflammatory cytokines through their receptor AT1R and BRB1.

Although the activation of the immune response is crucial to fight the infection, the overproduction of pro-inflammatory cytokines, also known as a “cytokine storm”, can lead to apoptosis and tissue damage, characteristics seen in patients critically ill with COVID-19 [28]. In fact, different works have demonstrated that the level of specific cytokines and chemokines is related to disease severity and these could be used as parameters to follow disease progression. For instance, Liang et al. demonstrated that, while IL-8 is constantly elevated in COVID-19 patients, IL-6 and IL-10 increase was correlated with the severity of the infection. The same was true for the chemokine Interferon-y protein 10 (IP-10) which is only transiently increased in the common influenza, while in COVID-19 patients its expression remains constant and, as with IL-6 and IL-10, is correlated with disease severity [29]. A mechanism that could explain an overproduction of pro-inflammatory cytokines or worsening of the infection is the dysregulation of the RAAS caused by SARS-CoV-2-dependent ACE2 internalization. Indeed, the accumulation of AngII due to a decreased ACE2 membrane level can cause vasoconstriction, the production of pro-inflammatory cytokines, and reactive oxygen species (ROS) generation through the overactivation of its receptor AT1R. In a recent work, it has been demonstrated that the infusion of AngII or an ACE2 blocker in swine can recapitulate several symptoms seen in COVID-19 patients, such as alveolar damage and increased coagulation [30]. It is important to note that ACE2 also plays an important role in the regulation of the KKS system. Indeed, it can inactivate [des-Arg9]-BK (DABK) and Lys-[des-Arg9]-BK (LDABK) that, through the BRB1 receptor, promotes pro-inflammatory cytokines production and angioedema [4] (Figure 5). It has been shown that levels of components of the complement pathways and the KKS were strongly correlated with severity and death in COVID19 patients [31] and some first evidence is indicating that KKS inhibitors could be beneficial in improving lung function in COVID19 patients [32].

Furthermore, it appears that ACE2 localization has a key role in determining disease severity. Critically ill COVID-19 patients show a pulmonary hypercoagulable state [4,28,33,34] and it has been suggested that the proximity of type II alveolar epithelial cells expressing ACE2 to the pulmonary microvasculature could be the reason for the diffuse thrombotic events caused by the hyper inflammation. The early assessment of biomarkers such as D-dimer, creatine kinase, and troponin T could help in the prediction and prevention of possible development of pulmonary intravascular coagulopathy [15].

## 6. Potential Treatments for COVID19: The Need to Find Multiple Therapeutic Options

The clinical reports published since the first outbreaks in December 2020 have indicated which part of the population is more at risk. In Table 2 we have summarized the findings of several studies, where advancing age [35], male sex, and the presence of comorbidities such as hypertension, diabetes, and cardiovascular disease seem to represent the main risk factors for severe outcomes [36]. Recently, obesity has been indicated as an additional risk factor, which can promote severe outcome in subjects infected with SARS-CoV-2. Kompaniyets and colleagues have reported a relationship between body mass index (BMI) and severe infection, probably due to the high level of inflammation characteristic of obese people [37].

In fact, after one year in this pandemic, although COVID19 has been shown to spread among young and middle-aged people, where only a small percentage develop severe symptoms [47,48], indicating that our efforts should be concentrated towards finding better therapies in subjects that are often treated for other pathologies. It is crucial to understand to what extent underlying diseases and their treatment could represent a risk factor for severe outcomes. Another important aspect to note is the risk of concomitant bacterial infection, which has previously been shown to exacerbate the symptoms of influenza viruses [49].

Today, there are no specific treatments for COVID-19 and all strategies being applied are entirely supportive [3], with many clinical trials underway that have not yet offered definitive support to any particular treatment. Since the beginning of this pandemic, different classes of drugs or treatments have been tested against SARS-CoV-2 infection, including anti-viral (AV) drugs, anti-inflammatory (AI), monoclonal antibody (MA), plasma therapy (PT) and cell-based therapy (CT).

**Figure 6:**
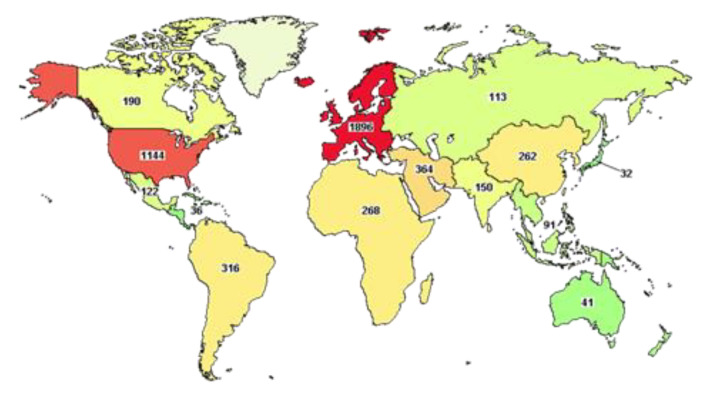
Map of COVID19 clinical trials. This map has been obtained by using clinicaltrial.gov searching for COVID-19 clinical trials for the age 65 and above.

At the time of writing, the website clinicaltrial.gov reports more than five thousand clinical trials underway worldwide (Figure 6), of which 980 have been completed.

An advanced search shows that half of these trials (2554) are focused on subjects older than 65 years, as discussed above, demonstrating the urgency of finding better therapies for the strata of the population that has suffered more from the effects of this pandemic. Among these trials, only 17 have been completed and they include the use of Ivermectin (AV), alone or in combination with Doxycycline, Hydroxychloroquine monotherapy (AV) and in combination with Azithromycin (AI), Favipavir (AV), Remdesivir (AV), Convalescent Plasma. Ivermectin is an FDA-approved AV drug, known since 1981 and proven to work against different RNA viruses, such as Avian influenza A, Zika, yellow fever, dengue, among others, and recently shown to completely block SARS-CoV-2 replication in vitro. This drug is normally well tolerated, with minimal side effects and, hopefully, when published, the results of these clinical trials will advise its use in COVID-19 subjects [50]. Other AV drugs, Chloroquine and Hydroxychloroquine, have been shown to inhibit viral entry, but the clinical reports published are vague and often present many limitations, such as an inadequate number of patients and no medical or safety outcome being described [51,52]. It is also worth remembering that both drugs have been shown to cause severe side effects, such as cardiotoxicity and hypoglycemia [53,54] that, as described previously, could cause harm in particular subjects with cardiovascular disease or diabetes who are more at risk of developing severe COVID-19 infection. Furthermore, more precautions should be used in subjects with Glucose-6-phosphate dehydrogenase deficiency [55]. Remdesivir (AV), proposed as a treatment for Ebola, blocks viral RNA replication through the inhibition of RdRp and has been demonstrated to control SARS-CoV-2 infection in vitro and in vivo [56]. The final report published on 1062 patients demonstrated that Remdesivir improved recovery in adults with COVID-19 and the decrease in mortality rate was statistically significant [57]. Like Remdesivir, Favipavir (AV) is an antiviral drug, able to inhibit the RdRp and, although the data of the current clinical trial have not been published yet, early indications have demonstrated a decreased viral load in COVID-19 patients [58]. Different studies have shown that plasma from convalescent patients (CP) can also improve the clinical outcome in severely ill patients [59], although this may not be the best therapeutic strategy when an extremely high number of patients needs to be treated. Beyond the molecules just described, many other repurposed FDA-approved drugs have been used to treat SARS-CoV-2 infections. For the sake of space, and because the description of the molecular mechanisms specific to each drug is beyond the scope of this review, we have listed them in Table 3 and refer the readers to other works, where their mode of action is described in detail [60].

## 7. A Race for a COVID19 Vaccine

Although some of these treatments are giving promising results, we are still far from having a standardized therapy protocol and, in many cases, the specific treatment and time of administration are crucial and can change among patients based on their health, age, and coexisting conditions. It is evident that a long-term strategy to fight SARS-CoV-2 infection is to reach herd immunity, with the administration of a specific vaccine to the entire world population. Since the publication of SARS-CoV-2 genetic sequences, the race for the development of a vaccine has interested both the private and public sectors. Multiple approaches have been applied, including the use of non-replicating viral vectors, messenger RNA (mRNA), inactivated whole-virus and DNA-based vaccines, all having the spike protein as a target [61]. These efforts have resulted in the development and production of different vaccines, some of which, such as Pfizer (BTN162b2) with an efficacy of 95% in preventing Covid-19 infection [62], Moderna with 90% mRNA 1273 and AstraZeneca with 70%, already being used to vaccinate millions of people. BTN162b2 and mRNA 1273 are mRNA-based SARS-CoV-2 vaccines, designed with an open reading frame of the spike protein with a polyadenylation signal at 3′ end, that stimulate humoral and cellular immune responses [63,64]. The mRNA is protected from degradation with warp in lipid nanoparticle. This lipid nanoparticle is not only a vehicle for the mRNA but is an adjuvant, increasing the T follicular helper and B cell of germinal centre response [64,65]. While this kind of vaccine can induce mild side effects, such as fatigue, headache, localized pain at the injection site and myalgia, the antibody response is high and long term, especially after the booster dose [66,67,68]. Another vaccination method is based on non-replicating adenoviral vectors. Adenoviruses (Ads) are an ideal vector candidate for SARS-CoV-2 vaccines due to their capacity to infect target cells and express transgene at a high level [69]. Furthermore, the Ads-mediated infection can induce the upregulation of costimulatory molecules that stimulate the cytokine and chemokine responses with a consequent improvement of the immune response [70]. The major disadvantage of Ads based vaccines is the pre-existing immunity against different Ads strains; in fact, Ads are common pathogens that cause a mild respiratory infection. Oxford University, in collaboration with AstraZeneca, has designed a chimpanzee adenovirus vector vaccine that expresses a whole spike protein, overriding the problem related to pre-existing immunity [71]. Generally, all vaccines based on non-replicating vectors are safe and immunogenic, inducing the production of anti-spike protein antibodies with mild side effects. Additionally, Janssen Pharmaceutical Companies (Johnson&Johnson) has developed a vaccine based on Ad26 vector encoding a full-length SARS-CoV-2 spike protein. A precise analysis reveals that the Ad26.COV2. S vaccine has an acceptable safety profile and its advantage with respect to the other vaccines is that this vaccine is immunogenic after one dose, with a local systemic reaction the day of immunization, or the following day, which resolves within 24 h [72]. In China, an inactivated variant of the SARS-CoV-2 virion produced in the Vero cells line is being used as a vaccine. The inactivation can be carried out using beta propiolactone and aluminiun hydroxide as an adjuvant. Alternatively, self-replicating RNA (saRNA) vaccines are based on a donkey Venezuelan equine virus strain (VEEV), in which the self-amplification coding region is conserved while the structural coding region is replaced with prefusion-Spike protein of SARS-CoV-2. In this case, the saRNA can be wrapped in various forms of nanoparticles [73]. There are also DNA-based vaccines under investigation based on a consensus SARS-CoV-2 spike protein sequence and, in addition, to improve immunogenicity, an N-terminal IgE leader is added. In this way, the interaction between the virus and the ACE2 receptor is blocked [74]. A summary of principal vaccines against SARS-CoV-2 is reported in Table 4.

## 8. Conclusions and Future Directions

The new pandemic caused by SARS-CoV-2 has made us more aware that coronaviruses are a threat that the scientific community must address. Indeed, the presence of a vast number of coronaviruses identified in bats, combined with their ability to accumulate mutations and rapidly change, makes future pandemics very likely. Many studies have confirmed that, as with previous SARS, SARS-CoV-2 uses ACE2 as a receptor for cell entry [1] although, as discussed above, increasing evidence is demonstrating that other receptors may be used by this new virus to enter the cells, which could explain its ability to infect multiple organs. Moreover, its increasing affinity to bind, due to a mutation in the Spike protein, has allowed it to rapidly spread in the population, causing severe pneumonia [75] and many deaths, especially among people suffering from hypertension, cardiovascular diseases [33,76], and diabetes. A potential reason for the high susceptibility of these subjects to develop severe conditions is the important role the ACE2 plays in different physiological pathways, such as the RAAS and KKS, among others. Indeed, the decreased level of membrane ACE2 due to SARS-CoV-2 dependent internalization can cause an increase in the level of both AngII and DABK and LDABK. AngII has been demonstrated to over-activate its receptor AT1R, causing proinflammatory cytokines production, vasoconstriction, caspase activation, and oxidative stress. In the same fashion, DABK and LDABK can exacerbate the inflammation by promoting proinflammatory cytokine release and angioedema through their receptor BRB1. Therefore, therapies that would target one pathway but not the other will not be as effective in decreasing the inflammation. The important role that ACE2 plays in different physiological pathways can also explain why therapies used to date have had a moderate success that is mostly dependent on medical history and the severity of the disease at the beginning of the treatment. There is no doubt that only through reaching the herd immunity can the fight against COVID19 be won. In fact, although with many difficulties, the vaccination campaign started in all countries around the world should decrease the number of infected people requiring hospitalization.

Some concerns have been raised regarding the efficacy of the currently available vaccines against the newly identified SARS-CoV-2 variants [11]. Although, as we have discussed above, many mutations would have occur on the spike protein to allow it to completely escape the immune response, is also true that the rate of SARS-CoV-2 mutations appears to be quite high, challenging the future efficacy of the available vaccine. Therefore, it is important to clearly understand the role that ACE2 and the other receptors used by SARS-CoV-2 play in maintaining organismal physiology. This will help us to identify the most effective therapeutic approaches based on the individual patient’s medical history.

## Figures and Tables

**Table 1:** Percentage of RdRp identity related to SARS-CoV-2 and different family of Coronavirus. Sequence reference are respectively YP_009725307, QHR63299.1, QDF43819.1, NP_828869.1, YP_009047223.1, AIW52769.1, YP_459941.1, AIW52828.1, YP_009555260.1.

SARS-CoV-2	Bat CoV	BtRs-BetaCoV/YN2018A	SARS -CoV	MERS	hCoV 229E	hCoV HKU1	hCoV NL63	hCoV OC43
	99%	96%	96%	70%	58%	67%	59%	66%

**Table 2:** Percentage of COVID19 patients with pre-existing conditions identified in the reported clinical studies.

Clinical Report	Nr Cases	Age	Males	Females	CVD	Diabetes	Hypertension
Zhonghua Liu Xing 2020 [38]	44672	30–69 (77.8%)	51.4%	48.6%	4.2%	1.1%	12.8%
Xie J et al., 2020 [39]	168	>50	75%	25%	18.5%	25%	50%
Guan WJ et al., 2020 [21]	1099	>50 (56%)	58.1%	41.9%	2.5%	7.4%	15%
Huang C et al., 2020 [40]	41	49 median	73%	27%	15%	20%	15%
Zhang JJ et al., 2020 [41]	140	57 median	50.7%	49.3%	not specified	12.1%	30%
Li Q et al., 2020 [42]	425	59 median	56%	44%		not specified	
Wang D et al., 2020 [43]	138	56 median	54.3%	45.7%	10.1%	14.5%	31.2%
Chen N et al., 2020 [44]	99	55.5 median	67.7%	32.3%	40%	13%	3%
Shi H et al., 2020 [45]	81	49.5 median	52%	48%	10%	12%	15%
Liang WH 2020 [46]	1590	48.9 median	57.3%	42.7%	3.7%	8.2%	16.9%

**Table 3:** Principal class of repurposed drugs involved into treatment of SARS-CoV-2.

Group	Drugs Name	Action
Anti-Inflammatory	1) Azithromycin	1) Immuno-modulatory effect;
	2) Tocilizumab	2) Humanized anti Il-6 receptor antibody, it bind soluble and membrane receptors blocking JAK-STAT pathways reducing inflammation;
	3) Corticosteroids	3) Helps dampens inflammation and other immune response;
	4) Thalidomide	4) Reduction of cytokine storm;
	5) Anakinra	5) Block IL-1;
	6) Rituxolitinib	6) JAK1 and JAK2 inhibitor;
	7) Bacitinib	7) Inhibits the kinase activities of JAK1 and JAK2.
Anti-Viral	1) Hydroxychloroquine;	1) Inhibit the virus entry into host cells increasing endosomal pH resulting in inhibition of membrane fusion between host cell and virus;
	2) Camostat;	2) Block viral maturation and entry into host cells;
	3) Remdesivir;	3) It terminates RNA synthesis and inhibits SARS-CoV-2 genome replication;
	4) Lopinavir;	4) Protease inhibitor, used in combination with ritonavir improving anti-viral activity;
	5) Ritonavir;	5) Used in combination with lopinavir;
	6) Favipiravir;	6) It is a guanine analogue, inhibits RNA polymerase;
	7) Umifenovir;	7) Inhibit viral and cellular membrane fusion;
	8) Ivermectin.	8) Block viral replication.
Monoclonal Antibody	1) Casirivimab;	1) Block viral entry into host cell;
	2) Imdevimab;	2) Block viral entry into host cell;
	3) Bamlanivimab.	3) Block viral entry into host cell.
Plasma Therapy	Immune serum (convalescent plasma)	Exploitation of virus-specific antibody
Cell- Based Therapy	1) Mesenchymal stem cell;	1) Ameliorate tissue regeneration;
	2) Natural Killer cell	2) Enhance immune response.

The abbreviations are as follow: Janus kinase/signal transducers and activators of transcription (JAK-STAT), Interleukin 1 (IL-1), Janus Kinase 1 (JAK1), Janus kinase 2 (JAK2).

**Table 4:** List of principal vaccines against SARS-CoV-2. In the table are indicated the type of vaccine such as messenger RNA (mRNA), self-replicating RNA (saRNA), Inactivate Virus, Non-Replicating Viral Vector, Viral Vector and DNA. Each type of vaccine is associated with its specific name and the name of the company.

	Name	Group	Clinical Status	Reference
mRNA	1) BNT162b2 and BNT162b1;	1) BioNTech/Fosun Pharma/Pfizer;	1) Recruiting;	(1) NCT04368728
	2) CVnCoV;	2) Curevac;	2) Recruiting;	(2) NCT04652102
	3) mRNA-1273.	3) Moderna/NIAID.	3) Recruiting;	(3) NCT04283461
saRNA	1) ARCT-021;	1) Arcturus/Duke-NUS;	1) Recruiting;	(1) NCT04480957
	2) LNP-nCoVsaRNA;	2) Imperial College London;	2) Phase I	(2) https://doi.org/10.1186/ISRCTN17072692;
Inactivate Virus	1) BBV152A/B;	1) Bharat Biotech;	1) Active;	(1) NCT04471519
	2) QazCovid-in®;	2) Research Institute for Biological safety Problems, Republic of Kazahstan;	2) Active;	(2) NCT04691908
	3) CoronaVac/PiCoVacc;	3) Wuhan Institute of Biological Products/Sinopharm	3) Active;	(3) NCT04456595
	4) Inactivated COVID-19 vaccine;	4) Wuhan Institute of Biological Products/Sinopharm	4) Phase III;	(4) ChiCTR2000034780;
	5) BBIBP-CorV;	5) Bejing Institute of Biological Products/Sinopharm	5) Enrolling by invitation;	(5) NCT04470609
	6) Inactivated SARS-CoV-2 vaccine;	6) Institute of Medical Biology, Chinese Academy of Medical Sciences	6) Recruiting;	(6) NCT04795414
	7) CoV-2 Vaccine.	7) Beijing Institute of Biological Products Co Ltd. China National Biotec Group Company Limited Fundación Huésped	7) Active.	(7) NCT04560881.
Non-Replicating Viral Vector	AZD1222 (ChAdOx1 nCoV-19);	University of Oxford/AstraZeneca	Active	NCT04516746
Viral Vector	1) Ad26.COV2·S;	1) Janssen Pharmaceutical Companies (Johnson&Johnson);	1) Active;	(1) NCT04505722
	2) Cansino Biological Inc./Bejing Institute of Biotechnology;	2) Ad5-nCoV;	2) Active;	(2) NCT04552366
	3) Gamaleya Research Institute.	3) Gam-COVID-Vac.	3) Active.	(3) NCT04564716.
DNA	1) AG0301-COVID19 and AG0302-COVID19;	1) Osaka University/AnGes/Takara Bio;	1) Active;	(1) NCT04463472
	2) INO-4800;	2) Inovio Pharmaceuticals/International Vaccine Institute;	2) Active;	(2) NCT04642638
	3) GX-19.	3) Genexine Consortium.	3) Recruiting.	(3) NCT04715997.

The abbreviation NIAD is used for National Institute of Allergy and Infectious Diseases. In the column clinical status is reported the current advancement of the clinical trial or if they are already been distributed to the population (Active). The references indicate the number of identifications on ClinicalTrials.gov that are identified with NCT followed by a number. In the case of Chinese registry are indicated with Chinese Clinical Trial Register (ChiCTR).

## Data Availability

Not applicable.
